# Dural fistula with bilateral arterial supply, mimicking a brainstem tumor

**DOI:** 10.1590/0100-3984.2015.0186

**Published:** 2017

**Authors:** Bárbara Liaffa, Fábio Noro, Paulo Roberto Valle Bahia, Flávia Pinto Dezonne Motta, Edson Marchiori

**Affiliations:** 1 Universidade Federal do Rio de Janeiro (UFRJ), Rio de Janeiro, RJ, Brasil.

**Figure 1:**
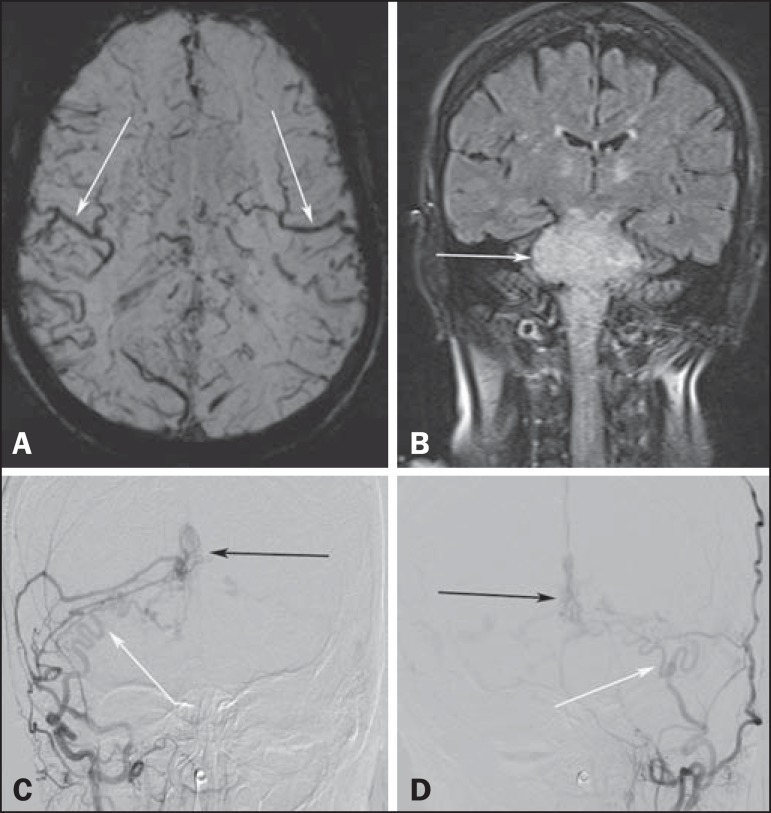
**A:** Axial slice in a susceptibility-weighted sequence showing numerous large-caliber superficial veins, representing venous congestion. **B:** Coronal slice in a fluid-attenuated inversion recovery sequence showing a hyperintense signal and increased brainstem volume, mimicking a brain tumor. **C,D:** Digital angiography with subtraction technique, revealing the bilateral nature of the arterial supply (white arrows) and the nidus (black arrows) formed by the fistula.

Dear Editor,

A 73-year-old woman presented with a history of at least four episodes of deep vein thrombosis. In the last five months, she had experienced severe ataxia, difficulty in swallowing, bilateral tinnitus, and symptoms related to intracranial hypertension, such as nausea and vomiting. Magnetic resonance imaging (MRI) revealed a hyperintense signal on T2-weighted images and an enlarged brainstem, the swelling extending to the thalamus, cerebellar peduncles, and to the cervical portion of the spinal cord (Figures 1A and 1B). The images could erroneously indicate a diagnosis of brainstem tumor, glioma in particular, due to the infiltrative pattern of the lesion and the increased organ volume. However, thorough evaluation with advanced imaging techniques, such as magnetic susceptibility-weighted sequences, demonstrated an extensive network of dilated peripheral veins, together with pronounced collateral circulation. Cerebral angiography showed a dural arteriovenous fistula (DAVF) with bilateral arterial supply via branches of the maxillary arteries. Venous drainage was mostly through the rectum and galenic system (Figures 1C and 1D). Involvement of the brainstem and cervical spinal cord was due to venous congestive injury. The classical surgical approach was precluded by the deep, inaccessible location, whereas endovascular therapy was precluded by the extensive involvement and bilateral nature of the fistula-sustaining arterial supply. The patient underwent gastrostomy and was discharged to palliative home care.

Vascular lesions are often difficult to diagnose^(1-8)^. DAVFs, which are characterized by abnormal communication between the arterial and venous systems, without intervening capillary beds, account for less than 10% of all cerebral vascular malformations^(9)^. The most common place of occurrence is the transverse sinus^(9)^, and there have been no reports of bilateral arterial supply. The two principal forms of presentation are hemorrhagic and non-hemorrhagic, both typically occurring as a consequence of intracranial venous hypertension^(9,10)^, which appears as the best predictor of poor prognosis^(11)^. Cerebral angiography continues to be the gold standard for the diagnosis of DAVF, in which the nidus represents the arteriovenous shunt itself and collateral vessels develop in order to drain the venous congestion^(12)^. Injury due to venous congestion is an example of a severe non-hemorrhagic manifestation, which can be prevented through the early diagnosis of DAVF, the treatment of choice being endovascular therapy, with the objective of interrupting the arterial supply to the venous system^(9)^.
